# EXAMINING PSYCHOSOCIAL FACTORS, HEALTH BEHAVIORS, AND WHITE MATTER LESIONS IN OLDER ADULTS

**DOI:** 10.1093/geroni/igz038.157

**Published:** 2019-11-08

**Authors:** Desiree Bygrave, Regina S Wright

**Affiliations:** University of Delaware, Newark, Delaware, United States

## Abstract

Prior to the onset of dementia, subclinical indices of brain pathology may reliably predict cognitive decline, even among older adults with high cognitive reserve. Evidence suggests that positive psychosocial experiences and healthy behaviors buffer cognitive decline. However, their relationship with brain outcomes in cognitively intact older adults is not well understood. Therefore, the current study examined the cross-sectional association between perceived social support, generalized anxiety, psychosocial stress, physical activity, sleep quality, and magnetic resonance imaging (MRI)-assessed white matter lesions (WML), among a diverse sample of older adults. We also examined sex and race as effect modifiers. Data were analyzed from 129 participants (mean age=67.40y, 69% female, 43% African American) enrolled in the Healthy Heart & Mind Study. Participants completed psychosocial and health behavior measures and MRI-assessed periventricular and deep WML were ascertained. Multiple regression analyses assessed relations of psychosocial responses and physical activity to WML, adjusting for known covariates. Significant general anxiety x sex interactions on deep WML (p<.05), significant physical activity x race interactions on total WML, frontal lobe WML and deep WML, respectively, and total sleep quality x race interactions on deep WML, were observed (p<.05). Conditional effects showed greater physical activity and sleep quality were associated with lower WML in African-American women; greater social belonging was associated with lower WML in American-American men; and lower anxiety was associated with lower WML in African-American women and White men. Results suggest positive psychosocial factors and health behaviors may influence subclinical brain pathology via unique pathways.

